# Intratonsillar detection of 27 distinct viruses: A cross‐sectional study

**DOI:** 10.1002/jmv.26245

**Published:** 2020-07-14

**Authors:** Antti Silvoniemi, Emilia Mikola, Lotta Ivaska, Marja Jeskanen, Eliisa Löyttyniemi, Tuomo Puhakka, Tytti Vuorinen, Tuomas Jartti

**Affiliations:** ^1^ Department of Otorhinolaryngology – Head and Neck Surgery Turku University Hospital and University of Turku Turku Finland; ^2^ Department of Clinical Microbiology, Turku University Hospital and Institute of Biomedicine University of Turku Turku Finland; ^3^ Department of Biostatistics University of Turku Turku Finland; ^4^ Department of Pediatrics and Adolescent Medicine Turku University Hospital and University of Turku Turku Finland

**Keywords:** human herpesvirus, parvovirus, polyoma virus, prevalence, respiratory virus, tonsil

## Abstract

Palatine tonsils have been observed to harbor several distinct respiratory and herpesviruses in separate studies. In this study, the presence of these viruses in palatine tonsils was comprehensively studied in both children and adults. A cross‐sectional analysis of 181 patients (median age 22 years; range, 2.6‐66) operated for a benign tonsillar disease was conducted. Real‐time polymerase chain reaction was performed to detect 27 distinct viruses in all: eight human herpesviruses, 16 respiratory viruses, parvo B19, and polyoma BK/JC viruses. Clinical characteristics of the patients and underlying conditions were evaluated. In total, 92% of patients had virus detected in tonsils (Epstein‐Barr virus 72%, human herpesvirus 7, and 6B 54% and 16%, respectively, enterovirus 18%, parvovirus B19 7% and the rest <4%). No herpes simplex virus 2, varicella zoster virus, polyoma JC virus, parainfluenza‐, metapneumo‐, or coronaviruses were found. Enterovirus was more common in children and was frequently observed in the presence of HHV6B. None of the viruses showed a positive association to the tonsillar disease. Respiratory symptoms were not associated with the prevalence of viruses. This study comprehensively reports a cross‐sectional view of intratonsillar virus infections in elective tonsillectomy patients in a wide age range cohort. Tonsils are a major virus reservoir for distinct herpes and respiratory viruses without a positive association with tonsillar disease or respiratory symptoms.

## Highlights

This study comprehensively reports a cross‐sectional view of intratonsillar virus infections in 181 elective tonsillectomy patients (median age 22 years, range 2.6–66). Enterovirus was more common in children and was frequently observed in the presence of HHV6B. Tonsils are a major virus reservoir for distinct herpes and respiratory viruses without a positive association with tonsillar disease or respiratory symptoms.

## 1 INTRODUCTION

Studying the prevalence of common pathogenic viruses in the tonsillar tissue of nonacutely ill patients has raised special interest for a variety of reasons. The tonsils act as a reservoir for persisting viruses (eg, herpesviruses), and reactivation of latent infection as well as viral shedding have been assumed to occur in the palatine tonsils.^1^, ^2^ The well‐known role of Epstein‐Barr virus (EBV) in nasopharyngeal malignancies and certain types of lymphomas maintains the interest of studying the interplay between this virus and the tonsillar tissue.^3^ Moreover, the palatine tonsils as secondary lymphoepithelial organs are essential in immune responses to respiratory microbes and allergic antigens. Thus, the tendencies of asymptomatic intratonsillar viral infections are of high importance since viral proteins are assumed to have significant effects on immunomodulatory reactions in tonsillar tissue.^4^ The interest of tonsillar tissue as an in vivo model for studying immunological reactions has recently generated a growing need for this kind of evaluation.^4^, ^5^

The palatine tonsils' tendency to harbor viruses asymptomatically has also led to suggestions that there might be a causal relationship between viral presence in tonsils and chronic adenotonsillar diseases, namely, tonsillar hypertrophy and recurrent or chronic tonsillitis.^6^ This is indeed interesting, as the etiology of these common diseases is still not completely understood. Nevertheless, conflicting results have been reported on these kinds of evaluations^6^, ^7^, ^8^, ^9^ and the lack of a healthy control group is a major drawback in virtually all of these studies.

Previous literature on viral prevalence in the tonsils is somehow fragmented since asymptomatic tonsillar viral infections have mainly been evaluated in protocols detecting single or a few viruses. Furthermore, very few studies have focused on tonsillar viral prevalence in adults, especially respiratory RNA viruses. In this study, the presence of multiple viruses was investigated in the tonsils of nonacutely ill patients. A setup of a large viral analysis panel was constructed, including the detection of eight herpesviruses, 16 respiratory viruses, parvovirus B19, and polyoma BK/JC viruses simultaneously.

## 2 PATIENTS AND METHODS

### 2.1 Patients

Human tonsil samples were consecutively obtained at Turku University Hospital in Turku, Finland between October 2013 and December 2014. Only nonacutely ill patients who underwent elective tonsillectomy due to a nonmalignant disease were eligible for the study. Patients were considered nonacutely ill, if they were free from respiratory symptoms both at the time of preoperative phone call a few days before surgery and on the operation day fulfilling the criteria for elective surgery. Exclusion criteria for the study were systemic use of anti‐inflammatory medication (other than nonsteroidal anti‐inflammatory drugs) within 4 weeks before surgery, suspicion of malignancy, and a chronic systemic disease affecting the immune system (such as an autoimmune disease, immune complex disease, or immunodeficiency). Nonetheless, patients with allergy, asthma, and atopic dermatitis were eligible. Written informed consent was obtained from all individual participants and/or their guardians. The study protocol was approved by the Ethics Committee of the Hospital District of Southwest Finland. All study procedures involving human participants were in accordance with the 1964 Helsinki Declaration and its later amendments.

### 2.2 Patient data

Participating patients were thoroughly interviewed for clinical information using a standard questionnaire, which included general health condition, medication, atopic diseases, current smoking habits, and respiratory symptoms (sore throat, fever, sneezing, coughing, and breathing difficulties) 2 weeks before surgery. Moreover, the indication of surgery was recorded at the time of operation.

The study patients were assessed in three age groups due to the assumed age‐related differences in tonsillar function and prevalence of chronic adenotonsillar diseases. Age group I consisted of pediatric patients (under 16 years old) and age group II of adolescents and young adults (16‐30 years) with immunologically active tonsils.^10^, ^11^ Age group III included patients over 30 years of age.

The patients were characterized to have tonsillar hypertrophy if their tonsils occupied more than 50% of the oropharyngeal airway, corresponding to a Brodsky score of 3 or 4.^12^ Chronic tonsillitis was diagnosed in patients who had had a sore throat and tonsillar inflammation for at least 3 months.^13^ Recurrent tonsillitis referred to at least four episodes of acute tonsillar infection in a year or three episodes in 6 months according to the Finnish guidelines of indications for elective tonsillectomy.^14^ In addition, a history of one or several peritonsillar abscesses was detected.

### 2.3 Sample collection, processing, and virus detection

Tonsillectomy was performed according to routine clinical practice. Immediately after removing the tonsils, several pieces of tonsillar core tissue were put into dry tubes and stored at −70°C for later analysis.

The nucleic acid extractions and polymerase chain reaction (PCR) tests were performed in the accredited clinical virus diagnostic laboratory according to standard daily protocols. The performance of nucleic acid extraction and PCR tests for human tissue samples have been analyzed earlier when applying the assays in clinical use. The analytical sensitivity of the *in‐house‐*tests were 5 to 10 cp/reaction, 1 to 5 cp/reaction for commercial multiplex herpesvirus test, and 50 cp/reaction for commercial multiplex respiratory virus test.

The tissue samples were homogenized and incubated with Proteinase K in +56°C for 1 hour before the nucleic acid extraction with NucliSens easyMag (bioMerieux, Lyon, France). Nucleic acids were stored at −70°C until analyzed. Rotor‐Gene 3000 instrument (Corbett Research/Qiagen) was used for PCR cycling, except for herpesviruses, whose detection was based on broad‐range PCR and microarray identification (Mobidiag, Espoo, Finland). The testing for entero‐ (EV), rhino‐ (RV), and respiratory syncytial (RSV) viruses were performed by an *in‐house* triplex PCR test.^15^ The primers for EV and RV were derived from the highly conserved 5′ noncoding regions of picornavirus genome are highly sensitive and eventually detect all entero‐ and rhinovirus genotypes.^16^, ^17^ F protein gene‐specific primers were used for RSV detection.^18^ Positive amplicons were identified as EV, RV, and RSV by melting curve analysis.^15^ Polyoma BK (BKPyV) and polyoma JC (JCPyV) viruses were analyzed using an in‐house PCR test as described earlier with slight modifications.^19^ The probe for BKPyV was 5′‐Cy5‐CAA CAC TCM ACA CCA CCC A‐MGB‐Eclipse‐3′. The amplification program included denaturation for 15 minutes at 95°C, and 40 cycles of 95°C for 15 seconds, 55°C for 30 seconds, and 72°C for 40 seconds. A duplex in‐house PCR test for testing human parvovirus B19 and human bocavirus was used. Briefly, the amplification reaction contained 5 µL of sample nucleic acids, 12.5 µL of Maxima probe PCR master mix, 400 nM of each primer (Parvo‐forward: 5′‐AGC AGT GGT GGT GAA AGC TC‐3′, Parvo‐reverse: 5′‐TTC CGA CAA ATG ATT CTC CTG‐3′, Boca‐forward: 5′‐GGA AGA GAC ACT GGC AGA CAA‐3′, Boca‐reverse 5′‐GGG TGT TCC TGA TGA TAT GAG C‐3′), and 100 nM of each probe (Parvo probe: 5′‐FaM‐CCC GCG CTC TAG TAC GCC CA‐BQ1‐3′, Boca probe: 5′‐Cy5 CTG CGG CTC CTG CTC CTG TGA T‐BQ2‐3′). The following instrument settings were used for amplification: +95°C for 15 minutes, 40 cycles of 95°C for 15 seconds, and 60°C for 60 seconds. Human herpesviruses (HSV1, HSV2, VZV, CMV, EBV, HHV6A, HHV6B, and HHV7) were analyzed using a commercial Prove‐It Herpes detection kit and Prove‐it TubeArray System (Mobidiag, Espoo, Finland) according to the manufacturer's instructions. Respiratory viruses were analyzed using Anyplex II RV16 detection kit (Seegene, Seoul, South Korea). The test detects the following viruses: adenovirus (AdV), human bocavirus 1 to 4 (HBoV), coronavirus (CoV) 229E, NL63, and OC43, EV, influenza A and B viruses (FluA and FluB), metapneumovirus (MPV), parainfluenza virus (PIV) types 1 to 4, RSV, and RV.

### 2.4 Statistical analysis

Continuous variables are presented as medians and ranges, and categorical variables as frequencies and percentages. The associations between categorical variables were evaluated using the *χ* ^2^ test. In cases of low‐frequency variables, Fisher's exact test was used. When assessing the associations between dependent variables (prevalence of viruses) and explanatory variables (age group, gender, smoking, prevalence of atopic diseases, respiratory symptoms, and surgical indications), logistic regression was performed and an odds ratio (OR) with a 95% confidence interval (95% CI) is expressed. Multivariable analyses for the prevalence of EV and HHV6B were performed with the same method including age group and surgical indication in the model. *P* < .05 (two‐tailed) was considered statistically significant. The statistical analyses were performed using SAS software version 9.4 (SAS Institute, Cary, NC).

## 3 RESULTS

### 3.1 Characteristics of the study population

In all, 183 patients participated in the study. One patient was excluded due to an incomplete clinical questionnaire and one patient due to technical problems in viral analyses. Consequently, 181 patients were eligible for the final analyses. The median age of the patients was 21.7 years (range, 2.6‐65.5), and the proportion of females was 56.4%. The characteristics of the patients and their clinical conditions are presented in Table 1.

**Table 1:** Patient characteristics and surgical indications

Total number (%)	Total	Male < 16 y	Female < 16 y	Total < 16 y	Male 16‐30 y	Female 16‐30 y	Total 16‐30 y	Male > 30 y	Female > 30 y	Total > 30 y
	181 (100.0%)	21 (11.6%)	16 (8.8%)	37 (20.4%)	36 (19.9%)	57 (31.5%)	93 (51.4%)	22 (12.2%)	29 (16.0%)	51 (28.2%)
Allergic rhinitis	70	3	2	5	17	22	39	12	14	26
Asthma	23	1	4	5	7	9	16	1	1	2
Atopic dermatitis	31	3	5	8	1	13	14	3	6	9
Current smoker	39	1	0	1	9	21	30	5	3	8
Surgical indications										
TH	25	14	8	22	1	0	1	1	1	2
CT	49	0	0	0	8	16	24	6	19	25
RT	27	0	2	2	7	14	21	0	4	4
PA	11	0	0	0	4	3	7	4	0	4
TH and CT	16	3	2	5	6	3	9	1	1	2
CT and RT	35	2	2	4	5	17	22	6	3	9
RT and PA	6	0	0	0	2	1	3	3	0	3
TH and RT	8	2	2	4	2	1	3	0	1	1
CT and PA	4	0	0	0	1	2	3	1	0	1

Abbreviations: CT, chronic tonsillitis; PA, history of peritonsillar abscess; RT, recurrent tonsillitis; TH, tonsillar hypertrophy.

Female patients reported more atopic dermatitis than male patients (*P* = .01). The prevalence rates of allergic rhinitis and asthma were higher in older patients than in younger ones (*P* < .001 and *P* = .044, respectively), and atopic diseases were more frequently observed in the presence of each other (data not shown). In addition, there was an association between smoking and age group (*P* < .001; Table 1). Otherwise, no associations were observed between age group, gender, atopic diseases, and smoking when assessing the relations between each other. Indications for surgery were different among the age groups (Table 1); tonsillar hypertrophy was more frequently present in the age group I, and the rest of the indications were more frequent in age groups II and III (*P* < .01 for each comparison between an individual indication and age groups). In addition, tonsillar hypertrophy and a history of peritonsillar abscess were more common in male patients (*P* = .004 and *P* = .006, respectively).

### 3.2 Symptoms before surgery

The rate of reported respiratory symptoms before surgery was related to the age of the patient (Table SA). Both sore throat (*P* = .037) and other respiratory symptoms (*P* = .021) were more frequently present in patients in age groups II and III than in the age group I. In addition, current smokers reported respiratory symptoms other than sore throat more frequently than those who did not smoke (*P* < .05), and female patients reported sore throat more frequently than the males (*P* < .05; Table SB). Otherwise, no associations were observed between the clinical characteristics and respiratory symptoms before surgery. Sore throat and other respiratory symptoms were associated with the presence of chronic tonsillitis (*P* < .001 and *P* = .018, respectively), whereas patients with tonsillar hypertrophy had fewer symptoms before surgery (*P* = .005).

### 3.3 Virus detection

**Figure 1:**
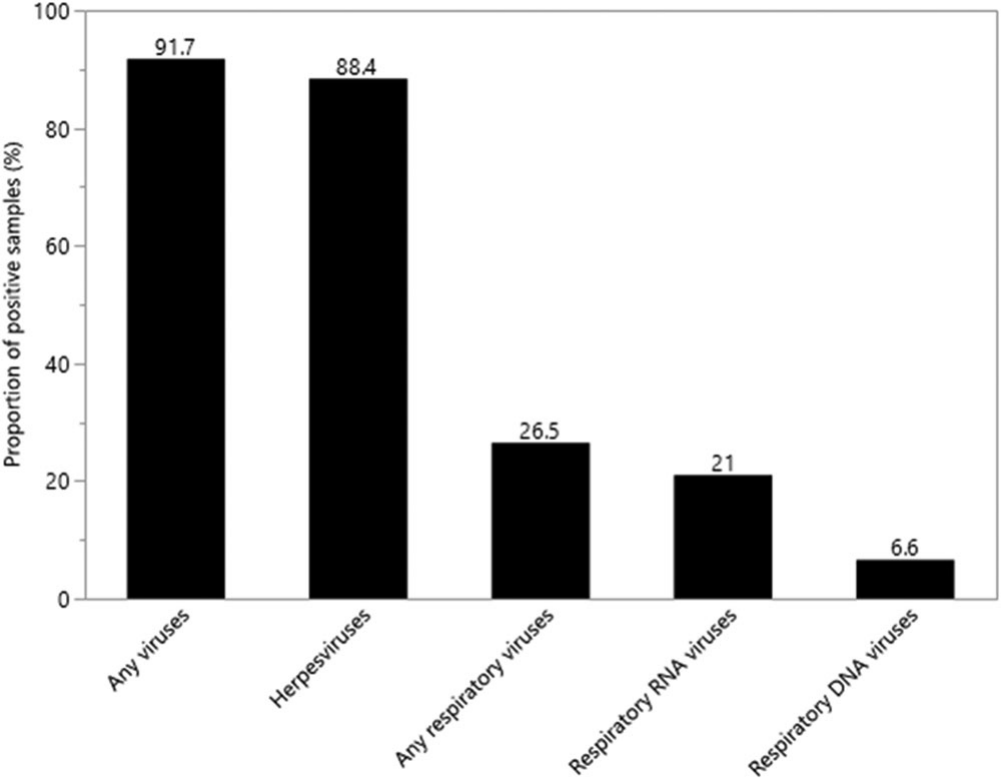
Frequencies of virus‐positive tonsillar samples in nonacutely ill tonsillectomy patients (n = 181)

The prevalence rates of all detected viruses are presented in Table 2. At least one intratonsillar virus was detected in 166 of 181 patients (91.7%). The most prevalent herpesvirus was EBV (71.8%), followed by HHV7 (53.6%) and HHV6B (16.0%). For respiratory viruses, EV had the highest prevalence (17.7%), followed by the DNA viruses HBoV (3.9%) and AdV (3.3%). Parvovirus B19 was observed in 7.2% and BKPyV in only 1.1% of patients. None of the patients had HSV2, VZV, PIV (1‐4), MPV, CoV (229E/NL63/OC43), or JCPyV in their tonsils. Cases of multiple infections are listed in Table SC and the frequencies of patients with herpesviruses and respiratory viruses in Figure 1.

**Table 2:** Intratonsillar viral prevalence in relation to patient characteristics and respiratory symptoms within two weeks before surgery^a^

	Total	No viruses	AdV	HBoV	EV	RSV^b^	RV	FluA	FluB	HSV1	CMV	EBV	HHV 6A	HHV 6B	HHV7	Parvo	BKPyV
Total number (%)	181 (100.0%)	15 (8.3%)	6 (3.3%)	7 (3.9%)	32 (17.7%)	5 (2.8%)	4 (2.2%)	2 (1.1%)	1 (0.6%)	1 (0.6%)	1 (0.6%)	130 (71.8%)	2 (1.1%)	29 (16.0%)	97 (53.6%)	13 (7.2%)	2 (1.1%)
Male	79	4	2	5	18	1	1	0	0	0	0	54	2	14	45	8	2
Female	102	11	4	2	14	4	3	2	1	1	1	76	0	15	52	5	0
<16 y	37	2	3	4	15	0	2	0	1	0	0	24	1	8	18	3	2
16‐30 y	93	9	3	1	9	1	1	2	0	1	0	67	0	15	50	9	0
>30 y	51	4	0	2	8	4	1	0	0	0	1	39	1	6	29	1	0
Asymptomatic	87	6	2	7	13	2	3	0	1	0	1	61	1	17	50	7	2
Sore throat	22	3	0	0	5	0	1	0	0	0	0	18	0	3	11	1	0
Other respiratory symptoms	39	3	3	0	7	2	0	0	0	1	0	25	0	4	18	4	0
Sore throat and other respiratory symptoms	33	3	1	0	7	1	0	2	0	0	0	26	1	5	18	1	0

Abbreviations: AdV, adenovirus; BKPyV, polyoma BK virus; CMV, human cytomegalovirus; EBV, Epstein‐Barr virus; EV, enterovirus; FluA/B, influenza A/B virus; HBoV, human bocavirus (1/2/3/4); HHV6A/6B/7, human herpesvirus 6A/6B/7; HSV1, herpes simplex virus 1; Parvo, parvovirus B19; RSV, respiratory syncytial virus; RV, rhinovirus (A/B/C).

a Herpes simplex virus 2, varicella zoster virus, human metapneumovirus, parainfluenza virus (1‐4), coronavirus (229E/NL63/OC43), and polyoma JC virus were not detected in any of the study patients.

b RSVA was detected in three patients, RSVB in one patient, and RSVA/B coinfection in one patient.

EV was detected more frequently in the presence of HHV6B infection (OR = 3.45, 95% CI, 1.27‐9.26, *P* = .014) when adjusted for age group and smoking. The association remained significant when adjusted for age group and the presence of chronic tonsillitis (OR = 3.76, 95% CI, 1.37‐10.2, *P* = .009). In the absence of EBV infection, parvovirus was detected more frequently, but the likelihood was not significant (OR = 0.31, 95% CI, 0.09‐1.01, *P* = .051). HHV6A (n = 2) was observed only in the presence of HHV6B infection.

### 3.4 Virus positivity in relation to clinical features

The results of virus detection in relation to age, gender, atopic diseases, and smoking are presented in Table 2 and Table SC. The prevalence of EV was related to the age of the patient (Tables 2 and 3), and the relationship remained significant when adjusted for smoking and surgical indications (*P* < .01). EBV infection was most frequently observed in age group III and was seen least in age group I, but the difference was not statistically significant (*P* = .49). Age group I had more respiratory DNA viruses (AdV and HBoV) than age groups II and III (Table 2), but the low numbers of cases hamper statistical assessment. Otherwise, no associations were observed between specific viruses and age, nor was the presence of any specific virus independently associated with gender, smoking, or presence of atopic diseases.

**Table 3:** Univariable and multivariable analyses of viral prevalence in relation to explanatory variables

	EV						HHV6B		
	Univariable			Multivariable			Univariable		
	OR	95% CI	*P* value	OR	95% CI	*P* value	OR	95% CI	*P* value
Age group II vs age group I	0.16	0.06‐0.40		0.20	0.07‐0.52		0.70	0.27‐1.89	
Age group III vs age group I	0.27	0.10‐0.73	<.001	0.30	0.11‐0.81	.003^a^	0.48	0.15‐1.53	.47
Sex (male vs female)	1.86	0.86‐4.07	.12				1.25	0.56‐2.78	.58
Allergic rhinitis	0.57	0.23‐1.27	.18				0.45	0.17‐1.07	.085
Asthma	0.98	0.27‐2.85	.97				0.76	0.17‐2.43	.68
Atopic dermatitis	1.46	0.53‐3.62	.43				1.01	0.32‐2.71	.99
Current smoker	0.20	0.03‐0.71	.034	0.33	0.05‐1.24	.15^b^	1.49	0.57‐3.57	.39
Any respiratory symptoms	1.44	0.67‐3.19	.35				0.60	0.26‐1.34	.22
Sore throat	1.48	0.65‐3.26	.34				0.85	0.33‐2.00	.72
Other respiratory symptoms	1.22	0.56‐2.64	.61				0.64	0.26‐1.45	.30
Tonsillar hypertrophy	2.53	1.13‐5.62	.022	0.82	0.22‐2.62	.75^c^	1.26	0.51–2.92	.60
Chronic tonsillitis	0.94	0.44‐2.06	.88	1.79	0.71‐4.90	.24^c^	0.39	0.17–0.87	.023^d^
Recurrent tonsillitis	0.93	0.42‐2.02	.86	1.57	0.66‐3.79	.31^c^	1.60	0.72–3.58	.25
History of peritonsillar abscess	0.46	0.07‐1.69	.31	0.82	0.12‐3.30	.80^c^	1.77	0.54–5.03	.31

Abbreviations: CI, confidence interval; EV, enterovirus; HHV6B, human herpesvirus 6B; OR, odds ratio.

a Adjusted for smoking.

b Adjusted for age group.

c Adjusted for age group and smoking.

d The prevalence of HHV6B was inversely related to the presence of chronic tonsillitis when adjusted for age group (OR = 0.41, 95% CI, 0.17‐0.97, *P* = .044).

HHV6B was less frequent in patients with chronic tonsillitis when adjusted for age group (OR = 0.41, 95% CI, 0.17‐0.97, *P* = .044). In addition, EV and HBoV were more frequent in patients with tonsillar hypertrophy (*P* = .02 for both), but the associations did not remain significant after adjusting for the age group. No other associations were observed between specific viral infections and indications of surgery (Table SD).

All patients with intratonsillar HBoV infection (n = 7) were asymptomatic before surgery. Conversely, FluA infection was observed in patients (n = 2), both of which had had respiratory symptoms. However, due to the low numbers of cases, statistical assessment was not possible in either observation. Comparing patients that reported any respiratory symptoms within 2 weeks before the surgery and those who did not, none of the individual investigated viruses were more prevalent in either group, excluding HBoV and FluA (Table 2). There was also no presence of any respiratory viruses, herpesviruses, or any number of all detected viruses in significant association to the presurgery symptoms.

## 4 DISCUSSION

This study was conducted to clarify the general view of multiple intratonsillar virus infections in nonacutely ill tonsillectomy patients. The study showed high intratonsillar prevalence rates for a few human herpesviruses, which was expected, but a reasonably low rate for respiratory viruses when compared with previous reports that were virtually limited only to the pediatric study populations. Respiratory symptoms 2 weeks before surgery were not associated with virus positivity. In addition, EV was frequently observed in the presence of HHV6B.

The study population was a pragmatic patient cohort undergoing routine tonsillectomy for a benign tonsillar disease at a single institution. Age group I, mainly consisting of patients undergoing operations for tonsillar hypertrophy, included more male patients than females, which is in line with the available corresponding data from the Swedish Tonsil Register.^20^ The reason for the male preponderance in the group of patients with a history of peritonsillar abscess remains obscure considering the epidemiological knowledge of the disease.^21^ However, the clinical characteristics of the study patients are in line with the general population undergoing elective tonsillectomy.

Human herpesvirus infections are commonly acquired early in life, and they are known to cause latent infections due to their capability to evade the host's immune system. In contrast to the other viruses in this study, the presence of HHVs have been under intense investigation in tonsil tissues. However, to our knowledge, this study is the second largest report regarding asymptomatic intratonsillar HHV infections. The prevalence of the most frequent herpesviruses, EBV and HHV7, were in line with the recently published SPLIT study, which reports a very large patient cohort investigated in 19 French hospitals.^22^ Conversely, the prevalence of HHV6B was remarkably lower in the present study than in the SPLIT study (16.0% vs 50.7%, respectively). On the other hand, the observed frequency of HHV6B was close to that (12.7%) reported in Italy a few years ago.^23^ The prevalence rates of EBV, HHV7, and HHV6B were not related to age, gender, or smoking, although the percentages of infected patients increased by age in EBV and HHV7 and decreased in HHV6B. These age‐related trends in prevalence rates are in line with the results of the SPLIT study.^22^ Other HHVs were observed in very few cases. This was probably the second study published to evaluate the prevalence of VZV in tonsils,^22^ but none of the patients were positive for VZV. There were no associations between respiratory symptoms and the presence of any of herpesviruses, which is not surprising considering the tendency of persistence/latency of HHVs.

The prevalence rates of intratonsillar respiratory viruses in the study population were reasonably low. Only 26.5% of patients had at least one respiratory virus in their tonsils. This is indeed interesting, since there are very few previous studies reporting corresponding data in a large age scale population. AdV is known to persist in mucosal T lymphocytes, and therefore, a higher frequency could have been expected to be observed. On the other hand, a previous study reported a peak incidence of AdV copies in adenoids and tonsils at 4 years of age and a decreasing trend after that with an apparent half‐life of 2.6 years.^2^ Moreover, in respiratory DNA viruses (AdV and HBoV), there has been a stronger tropism observed in adenoids than in palatine tonsils,^2^, ^6^, ^9^ and there is a higher prevalence of those viruses in pediatrics than in the adult population.^5^

Only elective tonsillectomy patients who were not acutely ill were eligible for this study. Meanwhile, mild respiratory symptoms within 2 weeks before surgery were not an exclusion criterion. Despite that, even lower rates of respiratory viruses were observed in tonsillar samples compared with some previous studies.^6^, ^9^ In Proenca‐Modena et al's^6^ study, only patients without symptoms 4 weeks before surgery had been included, and up to 68.6% of patients had at least one intratonsillar respiratory virus. In Faden et al's^9^ study, the corresponding rate of intratonsillar virus infection was 70.9%. This can be explained by the fact that only pediatric patients had been investigated in these previous studies. Probably because of the different indications for surgery in distinct age groups, the patients in the age group I had fewer respiratory symptoms before surgery compared with patients in age groups II and III. However, the youngest patients did not have fewer respiratory viruses in their tonsils than the older ones—the trend was rather opposite, although without statistical significance, even if the impact of EV was excluded (data not shown). In this study, only self‐reported history of respiratory symptoms was assessed, and additional analysis of respiratory viruses in nasopharyngeal swab might have provided completing data on the history of recent respiratory infections in the study patients. Indeed, previous studies have indicated that children have a tendency to present with asymptomatic viral shedding more frequently than adults.^24^ Accordingly, there might be a difference in the intratonsillar prevalence of all these viruses between pediatric and adult patients, and further studies are also needed to characterize these differences in respiratory RNA viruses.

Enteroviruses have been considered to be highly cytolytic, but it is well‐known that they are able to persist after acute infection in some tissues, such as the myocardium and pancreatic tissue.^25^ Only minor efforts have been made so far to study the intratonsillar prevalence of enteroviruses in asymptomatic adult patients. In this study, age was the strongest factor related to the prevalence of EV. It is noteworthy that no significant sex differences were observed in intratonsillar EV prevalence considering its higher general frequency and severe complications in males.^26^, ^27^, ^28^ Tonsillar hypertrophy was not independently associated with the prevalence of EV in contrast to a single previous report.^6^ Instead, EV was more frequently detected in the presence of HHV6B, which is a novel and interesting observation indicating a need for further investigation.

Parvovirus was detected in 7.2% of cases, which is a remarkably lower frequency compared with previous study populations reporting frequencies up to 16% to 45%.^29^, ^30^, ^31^, ^32^ However, methodological aspects to explain these differences cannot be ruled out considering the recent findings of parvovirus persistence in tonsils.^30^, ^31^ The differences in parvovirus persistence rates are likely due to differences in test sensitivity since parvovirus persists typically in very low copies.^33^ Only two patients (1.1%) were positive for the assay detecting BKPyV, which is comparable with previous corresponding studies using modern methods.^34^, ^35^

In this study, the only independent association between viral prevalence and indication for surgery was the inverse relation between HHV6B and chronic tonsillitis. This is an interesting preliminary observation since there seems to be a trend toward a decreasing prevalence of intratonsillar HHV6B near middle age,^22^ along with an increasing incidence of clinical manifestations due to cryptic chronic tonsillitis, such as tonsilloliths.^36^ The role of viruses in the pathogenesis of chronic adenotonsillar diseases has been speculated previously.^6^ However, it is explicitly challenging to draw any conclusions on the causality between tonsillar viral prevalence and chronic tonsillar diseases using this kind of study protocol. For ethical reasons, it is not possible to create an appropriate control group since tonsils cannot be obtained from healthy patients. Accordingly, observations are described in this context only in a descriptive manner.

There are some limitations in this study. Although a large panel of common pathogenic viruses were detected, no bacterial prevalence was investigated. Variations in the diversity of bacterial prevalence might contribute to changes in immunological responses in the tonsils, which also affects the presence of viruses. Furthermore, bacteria evidently play a major role in the pathogenesis of chronic tonsillar diseases, such as in the case of previously mentioned chronic tonsillitis. Then, in a study setup using a single time‐point detection of viruses, only limited assumptions can be made with regard to temporal dimensions of viral prevalence.

In future studies, the palatine tonsils can be used as an in vivo model in the field of immunological research. The rapid evolution of techniques in molecular biology will allow for the effortless verification of the entire intratonsillar virome. The present study highlights the relevance of this procedure since the complex nature of immunological responses may be affected by a high number of confounding factors, such as the presence of common pathogenic viruses in the tonsillar tissue. From a clinical point of view, detailed knowledge of tonsillar viral prevalence will probably promote the understanding of pathological processes associated with the function of secondary lymphoid tissues.

In conclusion, this study provides a comprehensive cross‐sectional report of intratonsillar virus infections in a pragmatic cohort of nonacutely ill tonsillectomy patients. Especially the data on respiratory RNA viruses in the adult population is mostly unique in its present form. None of the investigated viruses was positively associated with tonsillar diseases or respiratory symptoms before surgery. The tendency toward differences between intratonsillar viral prevalence in pediatric and adult populations, as well as the specific features of the prevalence of HHV6B and EV, indicate a need for further evaluation.

## CONFLICT OF INTERESTS

The authors declare that there are no conflict of interests.

## Supporting information

Supporting information
