# Supplementary material for: Intratonsillar detection of 27 distinct viruses: A cross‐sectional study
Source: J Med Virol. 2020 Jul 14;92(12):3830–8. doi: 10.1002/jmv.26245 (PMC7689766; doi:10.1002/jmv.26245)
Supplement: Supplementary file 1 — Supporting information [file JMV-92-3830-s001.pdf]

Supplementary Table A. Respiratory symptoms within two weeks prior to surgery in relation to surgical indications and age groups

	Total	Asymptomatic	Sore throat	Other respiratory symptoms	Sore throat and other respiratory symptoms
**TOTAL**	**181**	**87**	**22**	**39**	**33**
**TH**	**25**	**21**	**0**	**4**	**0**
< 16 years	22	18	0	4	0
16–30 years	1	1	0	0	0
> 30 years	2	2	0	0	0
**CT**	**49**	**17**	**11**	**8**	**13**
< 16 years	0	0	0	0	0
16–30 years	24	9	2	5	8
> 30 years	25	8	9	3	5
**RT**	**27**	**15**	**2**	**7**	**3**
< 16 years	2	1	0	1	0
16–30 years	21	11	2	5	3
> 30 years	4	3	0	1	0
**PA**	**11**	**6**	**1**	**2**	**2**
< 16 years	0	0	0	0	0
16–30 years	7	3	1	2	1
> 30 years	4	3	0	0	1
**TH+CT**	**16**	**6**	**4**	**2**	**4**
< 16 years	5	1	2	1	1
16–30 years	9	4	2	1	2
> 30 years	2	1	0	0	1
**CT+RT**	**35**	**14**	**2**	**12**	**7**
< 16 years	4	2	1	1	0
16–30 years	22	8	1	9	4
> 30 years	9	4	0	2	3
**RT+PA**	**6**	**2**	**0**	**2**	**2**
< 16 years	0	0	0	0	0
16–30 years	3	2	0	0	1
> 30 years	3	0	0	2	1
**TH+RT**	**8**	**5**	**2**	**1**	**0**
< 16 years	4	2	2	0	0
16–30 years	3	2	0	1	0
> 30 years	1	1	0	0	0
**CT+PA**	**4**	**1**	**0**	**1**	**2**
< 16 years	0	0	0	0	0
16–30 years	3	1	0	1	1
> 30 years	1	0	0	0	1

TH=tonsillar hypertrophy, CT=chronic tonsillitis, RT=recurrent tonsillitis, PA=history of peritonsillar abscess

Supplementary Table B. Respiratory symptoms two weeks prior to surgery and surgical indications in relation to gender, atopic diseases and smoking

	Total	Male	Female	Allergic rhinitis	Asthma	Atopic dermatitis	Current smoker
**TOTAL**	**181**	**79**	**102**	**70**	**23**	**31**	**39**
Asymptomatic	87	44	43	34	10	17	14
Sore throat	22	4	18	10	4	3	4
Other respiratory symptoms	39	17	22	14	3	5	14
Sore throat and other respiratory symptoms	33	14	19	12	6	6	7
TH	25	16	9	5	2	2	0
CT	49	14	35	29	8	9	6
RT	27	7	20	3	1	4	8
PA	11	8	3	4	1	0	3
TH+CT	16	10	6	7	2	4	3
CT+RT	35	13	22	14	6	9	12
RT+PA	6	5	1	4	1	1	3
TH+RT	8	4	4	2	2	1	3
CT+PA	4	2	2	2	0	1	1

TH=tonsillar hypertrophy, CT=chronic tonsillitis, RT=recurrent tonsillitis, PA=history of peritonsillar abscess

Supplementary table C. Viral prevalence in relation to atopic diseases and smoking

	**Total**	**Allergic Rhinitis**	**Asthma**	**Atopic Dermatitis**	**Current smoker**
**TOTAL**	**181**	**70**	**23**	**31**	**39**
No viruses	15	7	2	4	2
Single infection	51	17	6	9	14
Dual infection	76	34	12	9	20
Triple infection	27	9	3	7	2
Quadruple infection	11	3	0	2	1
Quintuple infection	1	0	0	0	0
AdV	6	1	0	3	0
HBoV	7	1	0	0	1
EV	32	9	4	7	2
RSV	5	4	1	1	0
HRV	4	3	1	2	0
FluA	2	2	1	0	0
FluB	1	0	0	1	0
HSV1	1	1	0	0	0
CMV	1	1	0	0	0
EBV	130	50	18	21	31
HHV6A	2	1	0	0	0
HHV6B	29	7	3	5	8
HHV7	97	40	10	14	18
Parvo	13	4	1	2	4
BKPyV	2	0	0	0	0

AdV=adenovirus, HBoV=human bocavirus (1/2/3/4), EV=enterovirus, RSV=respiratory syncytial virus, RV=rhinovirus (A/B/C), FluA/B=influenza A/B virus, HSV1=herpes simplex virus 1, CMV=human cytomegalovirus, EBV=Epstein-Barr virus, HHV6A/6B/7=human herpesvirus 6A/6B/7, Parvo=parvovirus B19, BKPyV=polyoma BK virus

Supplementary table D. Intratonsillar viral prevalence in relation to surgical indications and age groups

	Total	No viruses	AdV	HBoV	EV	RSV	RV	FluA	FluB	HSV1	CMV	EBV	HHV 6A	HHV 6B	HHV7	Parvo	BKPyV
**TOTAL (number and percentage)**	**181 100.0%**	**15 8.3%**	**6 3.3%**	**7 3.9%**	**32 17.7%**	**5 2.8%**	**4 2.2%**	**2 1.1%**	**1 0.6%**	**1 0.6%**	**1 0.6%**	**130 71.8%**	**2 1.1%**	**29 16.0%**	**97 53.5%**	**13 7.2%**	**2 1.1%**
**TH**	**25**	**1**	**2**	**4**	**9**	**1**	**1**	**0**	**0**	**0**	**0**	**16**	**1**	**5**	**14**	**2**	**2**
< 16 years	22	1	2	4	9	0	1	0	0	0	0	13	1	5	11	2	2
16–30 years	1	0	0	0	0	0	0	0	0	0	0	1	0	0	1	0	0
> 30 years	2	0	0	0	0	1	0	0	0	0	0	2	0	0	2	0	0
**CT**	**49**	**8**	**0**	**1**	**5**	**1**	**1**	**1**	**0**	**0**	**1**	**34**	**1**	**5**	**28**	**1**	**0**
< 16 years	0	0	0	0	0	0	0	0	0	0	0	0	0	0	0	0	0
16–30 years	24	4	0	0	0	1	0	1	0	0	0	16	0	2	14	0	0
> 30 years	25	4	0	1	5	0	1	0	0	0	1	18	1	3	14	1	0
**RT**	**27**	**1**	**2**	**1**	**2**	**0**	**0**	**1**	**1**	**0**	**0**	**20**	**0**	**6**	**17**	**1**	**0**
< 16 years	2	0	0	0	0	0	0	0	1	0	0	2	0	1	2	0	0
16–30 years	21	1	2	1	2	0	0	1	0	0	0	16	0	5	12	1	0
> 30 years	4	0	0	0	0	0	0	0	0	0	0	2	0	0	3	0	0
**PA**	**11**	**0**	**0**	**0**	**1**	**0**	**0**	**0**	**0**	**0**	**0**	**11**	**0**	**2**	**4**	**0**	**0**
< 16 years	0	0	0	0	0	0	0	0	0	0	0	0	0	0	0	0	0
16–30 years	7	0	0	0	1	0	0	0	0	0	0	7	0	2	3	0	0
> 30 years	4	0	0	0	0	0	0	0	0	0	0	4	0	0	1	0	0
**TH+CT**	**16**	**2**	**0**	**1**	**4**	**0**	**0**	**0**	**0**	**0**	**0**	**10**	**0**	**1**	**9**	**3**	**0**
< 16 years	5	1	0	0	3	0	0	0	0	0	0	3	0	1	1	1	0
16–30 years	9	1	0	0	1	0	0	0	0	0	0	6	0	0	6	2	0
> 30 years	2	0	0	1	0	0	0	0	0	0	0	1	0	0	2	0	0
**CT+RT**	**35**	**1**	**2**	**0**	**9**	**3**	**1**	**0**	**0**	**1**	**0**	**25**	**0**	**4**	**14**	**6**	**0**
< 16 years	4	0	1	0	2	0	1	0	0	0	0	2	0	0	2	0	0
16–30 years	22	1	1	0	4	0	0	0	0	1	0	15	0	4	9	6	0
> 30 years	9	0	0	0	3	3	0	0	0	0	0	8	0	0	3	0	0
**RT+PA**	**6**	**0**	**0**	**0**	**1**	**0**	**1**	**0**	**0**	**0**	**0**	**6**	**0**	**2**	**4**	**0**	**0**
< 16 years	0	0	0	0	0	0	0	0	0	0	0	0	0	0	0	0	0
16–30 years	3	0	0	0	1	0	1	0	0	0	0	3	0	0	2	0	0
> 30 years	3	0	0	0	0	0	0	0	0	0	0	3	0	2	2	0	0
**TH+RT**	**8**	**1**	**0**	**0**	**1**	**0**	**0**	**0**	**0**	**0**	**0**	**6**	**0**	**3**	**4**	**0**	**0**
< 16 years	4	0	0	0	1	0	0	0	0	0	0	4	0	1	2	0	0
16–30 years	3	1	0	0	0	0	0	0	0	0	0	1	0	1	1	0	0
> 30 years	1	0	0	0	0	0	0	0	0	0	0	1	0	1	1	0	0
**CT+PA**	**4**	**1**	**0**	**0**	**0**	**0**	**0**	**0**	**0**	**0**	**0**	**2**	**0**	**1**	**3**	**0**	**0**
< 16 years	0	0	0	0	0	0	0	0	0	0	0	0	0	0	0	0	0
16–30 years	3	1	0	0	0	0	0	0	0	0	0	2	0	1	2	0	0
> 30 years	1	0	0	0	0	0	0	0	0	0	0	0	0	0	1	0	0

TH=tonsillar hypertrophy, CT=chronic tonsillitis, RT=recurrent tonsillitis, PA=history of peritonsillar abscess. AdV=adenovirus, HBoV=human bocavirus (1/2/3/4), EV=enterovirus, RSV=respiratory syncytial virus, RV=rhinovirus (A/B/C), FluA/B=influenza A/B virus, HSV1=herpes simplex virus 1, CMV=human cytomegalovirus, EBV=Epstein-Barr virus, HHV6A/6B/7=human herpesvirus 6A/6B/7, Parvo=parvovirus B19, BKPyV=polyoma BK virus
